# Fernblock (*Polypodium leucotomos* Extract): Molecular Mechanisms and Pleiotropic Effects in Light-Related Skin Conditions, Photoaging and Skin Cancers, a Review

**DOI:** 10.3390/ijms17071026

**Published:** 2016-06-29

**Authors:** Concepcion Parrado, Marta Mascaraque, Yolanda Gilaberte, Angeles Juarranz, Salvador Gonzalez

**Affiliations:** 1 Pathology Department, School of Medicine, Universidad de Málaga, Malaga 29071, Spain; cparrado@uma.es; 2 Biology Department, Sciences School, Universidad Autónoma de Madrid, Madrid 28049, Spain; marta.mascaraque@estudiante.uam.es (M.M.); angeles.juarranz@uam.es (A.J.); 3 Dermatology Service, Hospital San Jorge, Huesca 22004, Spain; ygilaberte@salud.aragon.es; 4 Dermatology Service, Memorial Sloan-Kettering Cancer Center, New York, NY 10065, USA; 5 Medicine Department, Alcalá University, Madrid 28805, Spain

**Keywords:** *Polypodium leucotomos* extract, photoprotection, antioxidant, photoaging, oral, DNA, immunosuppression, photocarcinogenesis, ultraviolet (UV) radiation, visible light (VIS) radiation, infrared (IR)

## Abstract

Healthier life styles include increased outdoors time practicing sports and walking. This means increased exposure to the sun, leading to higher risk of sunburn, photoaging and skin cancer. In addition to topical barrier products, oral supplementations of various botanicals endowed with antioxidant activity are emerging as novel method of photoprotection. *Polypodium leucotomos* extract (PL, commercial name Fernblock^®^, IFC Group, Spain) is a powerful antioxidant due to its high content of phenolic compounds. PL is administered orally, with proven safety, and it can also be used topically. Its mechanisms include inhibition of the generation and release of reactive oxygen species (ROS) by ultraviolet (UV) light. It also prevents UV- and ROS-induced DNA damage with inhibition of AP1 and NF-κB and protection of natural antioxidant enzyme systems. At the cellular level, PL decreases cellular apoptosis and necrosis mediated UV and inhibits abnormal extracellular matrix remodeling. PL reduces inflammation, prevents immunosuppression, activates tumor suppressor p53 and inhibits UV-induced cyclooxygenase-2 (COX-2) enzyme expression. In agreement with increased p53 activity, PL decreased UV radiation-induced cell proliferation. PL also prevents common deletions mitochondrial DNA damage induced by UVA, and MMP-1 expression induced Visible Light and Infrared Radiation. These cellular and molecular effects are reflected in inhibitions of carcinogenesis and photoaging.

## 1. Introduction

Skin cancer accounts for at least 40% of all human malignancies. Solar radiation is a potent environmental human carcinogen, and its properties as a cancer-inducing agent are summarized in the *13th Report on Carcinogens* on 2 October 2014, of the National Institute of Environmental Health Sciences [1].

Ultraviolet (UV) radiation (UVR) is a major causal agent in most skin cancers [2]. Broad-spectrum UVR causes skin cancer by inducing DNA damage, increasing oxidative stress, suppressing the immune system response and promoting tumor growth through, among other mechanisms, mutation of the p53 tumor-suppressor gene [3,4]. UVR causes mutations in human cells and the type of lesion depends on the specific wavelength of UVR and whether cells can repair the lesion without error [1].

The use of botanical supplements equipped with significant antioxidant activity has generated great interest to decrease the risk of disease of the skin induced by UV radiation [5]. Polyphenols have been considered as a group natural bioactive products with potential health benefits. [6]. Plant extracts, herbs and spices containing these compounds have been administered for different diseases for thousands of years [7,8]. Today, plant polyphenols are being increasingly acknowledged by the scientific community for their added value as health boosters [7,8]. Currently, studies using plant polyphenols have proliferated, including 226 clinical trials that have been approved by the National Institutes of Health (NIH). At the moment of writing this paper, 35 open studies aimed at addressing the beneficial effects of polyphenols in health and diseases are recruiting participants.

In this context, *Polypodium leucotomos* (PL, commercial name Fernblock^®^, IFC Group, Spain) contains several hydrophilic extracts in its aerial part and these extracts exhibit powerful photoprotective properties after its administration, either topically or orally. *Polypodium leucotomos* (PL) is rich in phenolic compounds. PL contains among other cinnamic, ferulic and chlorogenic acids, all of them polyphenols with known antioxidant properties [9,10]. In vitro and in vivo studies have demonstrated that PL exerts its photoprotective by several mechanisms including inhibition of the generation and release of reactive oxygen species (ROS). It also prevents damage to the DNA, lipid peroxidation, activation of pro-inflammatory factors and induction of nitric oxide. It inhibits UV-mediated loss of cell-extracellular matrix adhesion, actin disarray and also prevents keratinocyte apoptosis [11]. In this review, we describe the composition of the extract and its antioxidant beneficial properties. To substantiate the molecular basis of the impact of oral PL for the prevention of the adverse cutaneous effects of UVR, we will summarize the state of the art of the molecular mechanisms and targets of PL involved in photodamage and skin cancer prevention. Briefly, these include: (i) signaling pathways that induce or regulate ROS generation and/or effects; (ii) pathways of DNA damage and DNA repair; (iii) role of ROS in inflammation; (iv) immune evasion of due to oxidative stress; (v) UV-induced tumor progression; (vi) interaction with UVR extracellular matrix (ECM) damage; and (vii) PL use in other non skin malignancies.

## 2. Solar Radiation and Deleterious Cutaneous Effects

The electromagnetic radiation emitted by the sun comprises ultraviolet radiation (UVR; 200–400 nm), visible light (VIS; 400–780 nm), and infrared (IR; 780 nm–1 mm). The International Commission of L’Eclairage (CIE) [12] classifies UV radiation into three types: UVA (315–400 nm), UVB (280–315 nm) and UVC (100–280 nm). UVC is completely filtered in the upper layers of the atmosphere. Although if a very limited exposure to UVA and UVB photons is necessary [13], excessive sun exposure is harmful to the skin and can be a cause of skin disease. Skin cancer induced by UV radiation takes many years to develop [4]. UV radiation produces photoaging and photocarcinogenesis through the generation of ROS, DNA damage, immunosuppression, inflammation and abnormal remodeling of ECM/angiogenesis [4].

Photoprotection is a practical approach to prevent skin cancers. In recent years, natural agents endowed with antioxidant, anti-inflammatory, anti-carcinogenic and immunomodulatory properties have garnered considerable interest due to their ability to prevent UV radiation-induced skin damage [14]. In the literature, UVR has been described as the main cause of the development of several skin pathologies. Intriguingly, great numbers of studies have also demonstrated radical species production after exposure to VIS and IR radiation [15]. The VIS part of the spectrum has not been as thoroughly investigated as the UV radiation subset [16,17]. VIS induces transient [18] as well as long lasting pigmentation in humans [19]. However, limited information is available regarding the side effect of VIS. Recently, several research studies showed that VIS induces significant ROS production thereby contributing premature skin photoaging [15,16,17,20].

IR radiation has both beneficial and negative effects on the skin [21], thus IR has found applications in the treatment of skin pathologies [21]; however, cutaneous exposure to IR radiation may affect the skin promoting ROS generation, interfering with mitochondrial functions and by generation of heat and mobilizing heat sensors at the skin surface [15,20,22].

### 2.1. Ultraviolet Radiation (UVR) and Photoinduced-Skin Cancer Aging

#### 2.1.1. Photooxidative Stress: Generation of Reactive Oxygen Species (ROS)

The main effects of UV radiation are DNA damage, immunosuppression and inflammation [23].

UVR induces the formation of ROS in the skin [23]. Oxidative stress is the principal cause of premature aging (“photoaging”). Oxidative stress is involved in several diseases, including cancer. At a cellular level, ROS generated in response to UV-R irradiation can activate cell surface receptors that activated mitogen-activated protein kinases (MAPK) [24,25]. The activator protein (AP-1) and the nuclear factor (NF)-κB are involved in the processes of cell proliferation, cell death, and cell survival. In addition, NF-κB is a key regulator of inflammation, oncogenesis, and apoptosis [26].

The inflammatory effect of UVR is due to induction of the oxidized membrane lipids to produce arachidonic acid released that is converted by cyclooxygenase enzymes (COX) into prostaglandins (PG), amplifying the recruitment of inflammatory cells to the area [27]. UVR activates AP-1, which contributes to photocarcinogenesis and the damage on ECM. AP-1 interferes with synthesis collagens I and III blocking the effect of transforming growth factor-β (TGF-β). TGF-β enhances collagen gene transcription. In addition, activation of AP-1 by UV irradiation causes overexpression of matrix metalloproteinases (MMPs) in human skin and extracellular matrix degradation. NF-κB regulation can also be modified by the activation of AP-1 [28].

#### 2.1.2. DNA Photodamage

UV radiation, mostly UVB, alters DNA by stimulation of the formation of specific DNA photoproducts, e.g., thymine-thymine dimers and pyrimidine-pyrimidine dimers. In addition, UVR damages telomeres, due to their elevated TT and G bases proportion. Thymine-thymine dimers are extremely important, mainly when they altered the tumor suppressor gene p53. In this regard, p53 mutations make cells resistant to apoptosis, with cells entering mitosis without having undergone DNA repair [29]. UV-B radiation also damages DNA through ROS. These may promote the generation of 8-hydroxy-2′-deoxyguanosine (8-OH-dG), a marker of DNA oxidative damage [30]. ROS damage genomic DNA, giving rise to mutations in critical genes that may lead to cancer development [29].

Finally, UV effect on mitochondria induces mtDNA “common deletion”, increasing ROS and a decreased cellular ability to generate energy [31].

#### 2.1.3. Photoinflammation

Acute and chronic exposure to UVR causes skin cancer through oxidation and inflammatory reactions. Acute UVR in the skin leads to erythema, edema, and inflammation [32]. Edema is due to excess leakage from hyperpermeable blood vessels by leukocyte infiltration, resulting in inflammation. In addition, transcription factors that control the inflammatory response such as NF-κB play critical roles in the pathogenesis of UVB-induced inflammation and carcinogenesis [33]. UV-induced ROS up-regulate the expression of the cyclooxygenase-2 enzyme (COX-2) [34]. COX-2 and prostaglandin E2 (PGE2) production contribute to the earliest stages of inflammation. In addition, other pro-inflammatory cytokines actively contribute to inflammation, such as interleukin-6 (IL-6) and tumor necrosis factor-α (TNF-α) [35]. Other pro-inflammatory mechanisms of UV radiation include ROS-induced peroxidation, which damages cellular membranes and induces activation of different isoforms of nitric oxide synthase (iNOS). UV-induction of iNOS seems to mediate VEGF-induced angiogenesis and hyperpermeability [36].

#### 2.1.4. Photoimmunosuppression

UV radiation induces immunosuppression, anergy and immunological tolerance. This is mediated by a marked decrease of the numbers of epidermal Langerhans cells (eLC), which leads to T helper 1 lymphocyte (Th1) clonal anergy [37]. The essential mechanism implicated in this process is the isomerization of the urocanic acid [3-(1*H*-imidazol-4-yl)-2-propenoic acid; UCA]. The isomerization of *trans*-UCA to the *cis*-isomer converts UV radiation into a biologically active identifiable signal that activates immune suppression [38].

In addition to alterations in antigen presentation and processing, the immunosuppressive effects of UV radiation are mediated by immunomodulatory molecules. These include both pro-inflammatory and anti-inflammatory molecules, such as prostaglandin PGE2, TNF-α and IL-10 [39].

### 2.2. Solar Non-Ultraviolet (UV) (Visible and Infrared) Radiation and Photoaging

UVA and UVB radiation have been the main focus of research on photoaging, but recent studies indicate a possible role for IR and VIS radiation in the pathogenesis of photoaging [16].

#### 2.2.1. Infrared (IR) Radiation

The photon energy of IR is much lower than that of UV. The largest part of solar IR radiation is IR A (IRA). IRA deeply penetrates into human skin but IR B (IRB) and IR C (IRC) only affect the upper layers of the skin [40,41]. Near-infrared (NIR) radiation (IRA: 760–1440 nm and IRB: 1440–3000 nm) and high doses of IRA radiation can produce deleterious effects in the human skin, but low doses of NIR radiation are indicated for the management of different cutaneous processes. In human skin, doses therapeutically acceptable lie in the range between 1 and 10 J/cm^2^. In this regard, low doses of NIR radiation has been used in the treatment of several diseases as superficial wounds healing, rheumatoid arthritis and Achilles tendinitis. Dermal fibroblasts are the main cell responsible for the beneficial action of IR radiation [21]. The molecular basis of the therapeutics effects includes: activation of mitochondrial cytochrome c oxidase; change the redox state of mitochondria cell membrane and activation the formation of NF-κB; increase expression of TGF-β1 and extracellular matrix proteins mediated by NF-κB; stimulation of reparative processes by extracellular matrix proteins and stimulation of fibroblast activity mediated by TGF-β1 activation [21].

IR radiation, however, can also have deleterious effects on the skin. A possible role for IR radiation in the pathogenesis of photoaging was proposed as early as 1982 by Kligman et al. [42]. More recent studies indicate that IR irradiation generates free radicals in human skin [43,44,45]. It is also known that IR radiation depletes the levels of beta-carotene and lycopene in human skin that only can be explained by the action of free radicals. Concerning the molecular mechanisms IRA-induced photoaging, mitochondrial ROS generation was shown to be the initiating event [46] followed by increased MMP-1 via activation of the MAPK, specifically extracellular signal-regulated kinase (ERK), c-JUN N-terminal kinase (JNK), and p38 kinase [42]. It was also shown that IRA leads to an overexpression of MMP-1 without an increase in expression tissue inhibitor of metalloproteinase (TIMP) [47], and increased also MMP-9 [48]. Finally, IRA seems to be confined to the dermal compartment and decreases the synthesis of the collagen itself. IR, as described before, induces similar effects to UV, but the essential mechanisms implicated in their actions are substantially different.

Besides the effect on metalloproteases, IRA produces other cellular responses, e.g., angiogenesis and increased the number of mast cells, both events associated with skin aging [49,50]. However, IRA decreases the cell turnover of keratinocytes and Langerhans cell density, influencing wound repair and altering the levels of transforming growth factor beta (TGB-β) [51].

#### 2.2.2. Visible Light (VIS) Radiation

Until recently, VIS (400–700 nm) has been regarded to have no significant cutaneous photobiologic effects. This was not entirely true, as an early study from Pathak et al. [18]. Pathak in 1962, reported that exposure to VIS results in immediate pigment-darkening (IPD) [18]. In 1984, Kollias and Baqer [19] performed an in vivo study to observe the pigmentary induced by VIS and NIR light. They reported that the pigmentation may occur without a significant UV component. This led to the hypothesis that VIS could contribute to photoaging and pigmentary conditions. This has been proven formally in recent years [16,20].

VIS contributes to ROS production in the skin [17]. Exposure of human skin to increased doses of VIS, from 40 to 180 J·cm^−2^, resulted increase in ROS production in a dose-dependent manner, similar to UV [20]. VIS produces DNA damage indirectly through the generation of ROS. It has been verified in melanoma cells and fibroblasts that there was no correlation between cyclobutane pyrimidine dimers (CPD) production and micronucleus formation induced by VIS [52].

The effect of VIS in the ECM is similar to that of UVR, IR plus VIS increased MMP-1 and MMP-9 expression and decrease type I procollagen levels in human skin in vivo, and recruited macrophages to the irradiated site [53]. Along with ROS generation by VIS, it has recently been proven that visible blue-violet light induces free radicals in human skin in vivo. Using a radiation source of blue-violet light, the concentration of skin carotenoids was measured noninvasively using resonance Raman spectroscopy over a period of 24 h after irradiation. Irradiation of human skin with blue-violet light induces significant dose-dependent degradation of carotenoids. This degradation of carotenoids reflects how blue-violet light generated free radicals and ROS in skin. In all individuals tested, the concentration of skin carotenoids declined in a similar manner as that caused by the infrared or ultraviolet radiation, leading to the conclusion that also the blue-violet light at high doses could cause damage equivalent to UV and IR radiation on human skin [54]. Regarding pigment darkening, the early studies described above have been confirmed late. In fact, recent studies reported that the immediate pigment darkening (IPD) induced by VIS is not significantly different from that produced by UV fraction suggesting that both UV and VIS interact with the same precursor [55]. Also in relation to UV radiation, both UVA and VIS can induce pigmentation in skin types IV–VI but the pigmentation induced by VIS was darker and more sustained [56]. These findings have potential implications and clinical relevance because the possible role of VIS in the pathogenesis of photo-induced pigmentary disorders, such as melasma or post inflammatory hyperpigmentation [16].

Finally, although sunscreens protect against UV photons efficiently, they are not as efficient stopping VIS and IR photons. Thus, the levels of skin ROS may overcome the natural antioxidant reservoir in the skin and cause skin damage [14,20]. This line of reasoning underlies therapies aimed at increasing the antioxidant threshold of the skin, as they seem to effectively increase its buffering ability against ROS induced by solar radiation [51].

## 3. *Polypodium leucotomos* (Fernblock^®^) as Photoprotective Agents

*Polypodium leucotomos* (PL) (synonyms *Phlebodium aureum*) is a fern of the Polypodiaceae family, genus *Phlebodium*, native to Central and South America. It has been traditionally used for treating skin diseases (e.g., psoriasis and atopic dermatitis) in the original areas [57].

The interest in the mechanism of action and overall properties of PL has spiked in recent years, mainly focusing on its antioxidant properties [58,59,60,61,62]. PL displays anti-inflammatory and immunoregulatory effects and also suppresses tumor growth [57]. It also accelerates the removal of UV-induced photoproducts, which contributes to its photoprotective effects [63,64,65,66,67,68].

Fernblock^®^ is controlled aqueous extract of the leaves of PL that has been developed to take advantage to take advantage of the photoprotective properties of ferns by providing a consistent phenolic content.

PL has been marketed in Europe since 2000, both in topical and oral formats and, presently, PL is commercialized in more than 26 countries, including the United States. In the United States, PL has been available as a dietary supplement since 2006 [69]. Studies on the pharmacology of Fernblock^®^ and their biological disposition have demonstrated that its toxicity is negligible even at high doses [70].

The photoprotective dose in healthy humans is 7.5 mg/kg [67,68]. PL, used topically, inhibited erythema at 0.1% (weight/volume) [71]. Its mechanisms of action and effects on successful clinical outcomes, and continued interest in the use of natural products such as polyphenols, have placed PL as an emerging trend as photoprotector and antioxidant [57,60].

### 3.1. Composition

The chemical composition of PL has identified the following compounds: p-coumaric, chlorogenic, caffeic, ferulic and vanillic acids. Phenolic compounds are secondary metabolites ubiquitously present in plants. In the human diet, polyphenols are the most abundant antioxidants [72,73] and they are present in beverages and foods of botanic origin. Moreover, they have many beneficial effects on human health. Phenolic compounds in the diet have beneficial effects in mammalian cells, including antioxidant activity, modulation of gene expression and inhibition of tumorigenesis in different models [73].

High-Performance Liquid Chromatography (HPLC) revealed that PL contains several phenolic compounds that were separated according to their retention time. The most abundant were phenolic acids (cinannamic acid), specifically 3-methoxy-4-hydroxycinnamic acids (ferulic), 4-hydroxycinnamic acid (p-coumaric), 3,4-dihydroxycinnamic acid (caffeic), 3-methoxy-4-hydroxybenzoic acid (vanillic), 3,4-dihydroxycinnamic acid (caffeic), and 3-caffeoilquinic acid (chlorogenic) [9]. To study whether these could be responsible for the beneficial effects of the extract, their absorption rate was studied in vitro. Their absorption of the phenolic compounds was studied using Caco-2 cells in order to resemble the intestinal barrier. Their antioxidative capacity was evaluated by the luminol/H_2_O_2_ assay. Finally, their metabolism was assessed using cultured primary rat hepatocytes. The most powerful antioxidants were ferulic and caffeic acids. The PL components increased the antioxidant capacity in a concentration-dependent manner. For all tested substances, the apparent permeability was similar to human post-oral administration absorption of 70%–100%. Coumaric, vanillic acids and ferulic acids were metabolized by CYP450-dependent mono-oxygenases. They were partially conjugated to sulfate and glucuronic acid [10].

### 3.2. Cellular and Molecular Evidence of the Photoprotective Properties of Fernblock^®^

PL extract constitutes the basis of Fernblock^®^. Oral intake of PL protects against the effect of UV-induced oxidative stress. Early studies described the anti-tumoral effect of PL extracts and others from similar ferns [74]. These results were confirmed in a hairless albino mouse model, in which topically applied PL inhibited skin tumor formation after UVB irradiation [75]. The anti-tumoral properties of PL likely reside in its protective effect against UV induced DNA damage [65,68]. In addition, PL blocked the effect of UV radiation on the expression of COX-2, which is an inducible enzyme responsible for prostaglandin synthesis that is also involved in carcinogenesis. Finally, PL induced activation of the tumor suppressor p53 and inhibits epidermal proliferation, decreasing the number of cyclin D1 and proliferating cell nuclear antigen (PCNA) positive cells induced by UVR [64,76].

Regarding the anti-inflammatory properties, in skin irradiated with UVB and UVA radiation, PL successfully blocked the inflammatory response elicited by of UV radiation [65,71]. PL decreased phototoxicity during Psoralens + UVA (PUVA) therapy [67,77]. Moreover, oral PL inhibited PUVA-induced infiltration of neutrophils, mast cells and formation sunburn cells, and inhibited the observed decrease of epidermal Langerhans cells (eLC) associated with these treatments. The beneficial effect of PL is probably due to decreased DNA damage and apoptosis, and correlates well with the reduction of skin photodamage [61].

In addition to its antioxidant activity, PL bears promise in the treatment and prevention of photoaging, due to its proven effects on extracellular matrix remodeling. PL inhibits several matrix MMPs, by inducing TIMP, elastin, TGF-β, and different types of collagen [78], thus promoting regeneration.

At a cellular level, PL prevents membrane damage and lipid peroxidation induced by UV. PL also blocks UV-mediated disarray of the actin cytoskeleton with the loss of cell–cell adhesion and cell–ECM adhesion [79]. Finally, PL inhibits keratinocyte and fibroblast cell death induced by UV radiation [66].

**Figure 1:**
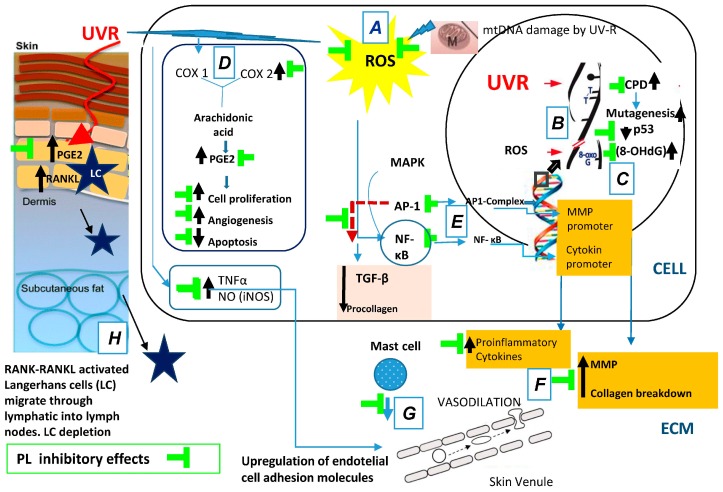
*Polypodium leucotomos* (PL) extract’s anti-UV effects: (**A**) decreases pro-inflammatory mechanisms of UV radiation include ROS-induced lipid peroxidation; (**B**) decreases UV-induced DNA damage; (**C**) increases the expression of active p53; (**D**) inhibits UV-induced Cox-2 enzyme levels; (**E**) reduces UV-induced nuclear transcription factors AP-1 and NF-κB; (**F**) reduces MMPs production; (**G**) decreases inflammation and vasodilation; and (**H**) inhibits skin immunodepression. Black arrows indicate increased/decreased effects due to UVR. ROS, reactive oxygen species; mtDNA, mitochondrial DNA; UV, ultraviolet; ECM, extracellular matrix; CPD, cyclobutane pyrimidine dimers; MMP, matrix metalloproteinase; MAPK, mitogen-activated protein kinases; AP-1, activator protein-1; NF-κB, nuclear factor kappa beta; TGF-β, transforming growth factor-β.; COX, cyclooxygenase enzymes; PGE2, prostaglandin E2; TNF-α, tumor necrosis factor-α; iNOS, isoforms of nitric oxide synthase; RANK, receptor activator of nuclear factor kappa-B; RANKL, receptor activator of nuclear factor kappa-B ligand.

In summary, oral supplementation of the natural antioxidant PL extract affords the following photoprotective effects: (a) decreased pro-inflammatory mechanisms of UV radiation include ROS-induced peroxidation; (b) decreased of UV-induced oxidative DNA damage; (c) increased in the expression of active p53; (d) inhibition of UV-induced COX-2 enzyme levels; (e) reduction of UV-induced nuclear transcription factors AP-1 and NF-κB; and (f) increase the levels of TIMP. All of these effects are related to the prevention of photoaging and photocarcinogenesis (Figure 1).

### 3.3. Fernblock in Photodamage and DNA Repair and Cellular Homeostasis

When administered orally, PL inhibits UV-mediated DNA damage and mutagenesis. It prevented the UV-induced accumulation of CPD [65,68,76]. This may be due to an improved function of the DNA repair systems, due to PL capacity to decreased oxidative damage [66].

In humans and in a xeroderma pigmentosum rodent model (XPC), PL inhibits the UV-mediated formation of thymine dimers [65,68]. XPC rodent model exhibit aggravates inflammatory response to UV irradiation and a reduction in their DNA repair capability. As a result, they have more risk to develop skin cancer.

PL also reduced systemic oxidative damage, as shown by the reduction of 8-OH-dG-positive cells. Orally supplementation with PL reduces the level of basal oxidative stress (quantified as a number of 8-OH-dG-positive cells), advancing that PL reduces constitutive oxidative DNA damage. 8-OH-dG is an important marker of early DNA damage and is mutagenic, favoring GC→TA mutations. In addition, PL decreased the levels of 8-OH-dG even before UV irradiation, suggesting that PL reduces constitutive oxidative DNA damage [65].

Finally, another clinical study has revealed that oral PL decreases UVA-dependent mitochondrial DNA damage by decreased common deletions (CD), which are mitochondrial markers of chronic UVA irradiation in fibroblasts and keratinocytes [31] (Table 1).

### 3.4. Fernblock Effects on Inflammation

Evidence of the role of PL in preventing UV-mediated inflammation stems from experiments that revealed that oral PL prevented erythema in UV-treated human skin [68,71,77] and also in PUVA-based therapy [67], which is often used in the treatment of vitiligo, psoriasis, and other inflammatory skin diseases [85,86]. The molecular basis of its anti-inflammatory properties could be due to its ability to suppress the expression of the following factors: (i) pro-inflammatory cytokines such as TNF-α and inducible NOS (iNOS) [35]; (ii) redox-sensitive transcriptional factors AP-1 and NF-κB [35,57,87]; and (iii) COX-2 and PGE2 [65].

Solar-simulated radiation (SSR) experiments in vitro showed that PL blocks the triggering of activation of activator protein 1 (AP-1) and NF-κB induced by UV radiation [35]. SSR-induced activation of AP1 and NF-κB in HaCaT cells, which was partially inhibited by PL treatment to the cells. This is a specific effect of PL that cannot be completely explained by to its antioxidant properties, as treatment of the cells with a bona fide antioxidant (AA) did not prevent the activation of AP-1 and NF-κB induced by solar-simulated radiation (SSR) [35]. However, some of the genes responsive to AP-1 are oxidative sources, including COX-2. UV-irradiated skin and tumors skin induced by UV radiation overexpress the inducible isoform COX-2, and COX-2 inhibition reduces photocarcinogenesis.

Importantly, PL inhibits expression of COX-2 induced by the UV-radiation [65]. COX-2 in vivo induces the synthesis of PGE2, which is a potent inducer of vasodilation. Inhibition of COX by PL decreased the presence of mast cells and leukocyte extravasation in the irradiated area [65,67,68]. PL also inhibits apoptosis and cell death [35,63] in vivo and in vitro thereby preventing apoptosis/necrosis-triggered inflammation.

The photoprotective effect of PL in SSR experiments with HaCaT cells supports the anti-inflammatory PL effects by counteracting SSR-dependent induction of TNF-α expression and production of nitric oxide (NO). PL decreases iNOS up-regulation induced by UV light [35]. This correlates well with inhibition of pro-inflammatory, UV-induced AP1 and NF-κB (Table 1).

A recent study addressed the effect of PL supplementation in the inflammatory reaction of the skin and tanning following a single exposure to solar-simulated radiation (SSR). A daily oral supply of PL reduced the inflammatory reaction induced by a single exposure to SSR with a significant increase erythematosus threshold. Conversely, it had no effect on the pigmentary reaction. Evaluation of changes in pigmentation in vivo should also be noted, because antioxidants reduce the phototoxic damage and subsequent elimination of keratinocytes of the upper epidermal layers, leading to longer retention of these cells and their skin melanosomes [88].

In conclusion, oral PL supplementation reduces the phototoxic erythematous reaction and if super-erythemogenic dose is delivered, it enhances skin recovery. All these activities likely play an important role in the acute and chronic harmful effects of sun exposure (Table 1).

### 3.5. Fernblock and Immunosuppression

Skin immune suppression is caused by the uncontrolled overexposure to UVR. Preventive measures, including photoprotection, are helpful. PL has shown immunomodulatory properties [89]. PL is as an oral antioxidant and photoimmunoprotective agent by several mechanisms: (a) interfering with *cis*-UCA isomerization [81]; (b) inhibiting of pro-inflammatory cytokines such as TNF-α [35]; (c) anti-apoptotic effects on dendritic cells (DCs) and eLC; and [67,68]; (d) inhibiting glutathione oxidation [66,77,80].

Elimination of eLC after UV radiation is induced by direct cell apoptosis, inflammatory phenomena, the aberrant morphology of eLC, and inhibition of adhesion molecules that are necessary for DCs migration from the epidermis to the deep skin layers. Efficiently, PL in vivo prevents eLC depletion produced by UV irradiation as well as the appearance of abnormal DCs [66,67,68].

Similar results were obtained irradiating blood dendritic cells using a solar simulator. UV radiation-induced a reduced expression of molecules activating for antigen capture. On the other hand, solar-simulated radiation produces overexpression of TNF-α and IL-10 by DCs, but no IL-12. Pretreatment with PL partially in vivo blocked secretion of these cytokines by UVR and consistently revealed higher numbers of LCs in PL-treated, the irradiated group [60]. Multiple molecular mechanisms underlie enhanced DCs survival, including in vitro inhibition of *trans*-UCA isomerization [81] and blockade in vitro of iNOS expression induced by UV radiation [30], which generates altered nitrogen oxide metabolites that cause immunosuppression by eliminating skin DCs and enhancement of endogenous systemic antioxidant systems that lead to decreased oxidized intermediates, e.g., oxidized glutathione [66] (Table 1).

### 3.6. Fernblock, an Anti-UV-Induced Tumor Progression Agent

Several research works have reported the anti-tumor effects of different fern extracts, including PL [57,87]. In this regard, in the hairless albino mouse model, PL, topically applied, blocked skin tumor formation and photoaging resulting from exposure to UVB radiation, even after discontinuation of the treatment for eight weeks [75]. In addition to blocking ROS generation efficiently, PL has shown anti-mutagenic properties and also cell protection ability from immortalized mutations preventing carcinogenesis [65]. Furthermore, PL induced activation of p53 [59,65]. In agreement with increased p53 activity, PL also decreased UV radiation-induced cell proliferation [68]. In agreement with inhibition of epidermal proliferation, PL decreases the number of cyclin D1 and PCNA positive epidermal cells induced by UVR [76]. As outlined above, PL inhibits the overexpression of COX-2 induced by UV radiation [65,76], which is also involved in carcinogenesis.

PL enhanced both p53 expression and activation in irradiated XPC (Xpc^(+/−)^) mice [65]. Activation of p53 has been shown to decrease the expression of COX-2, thereby reducing the inflammatory response [65]. Levels of phospho-p53 Serine 15, presumably an active form of p53, were increased in the skin of PL-treated mice, which inversely correlated with decreased COX-2 levels, suggesting that orally administered PL reduces UV-induced COX-2 levels in mouse skin through, at least in part, by activating tumor suppressor protein p53. Previous in vivo studies reported that PL increased p53 in UV-irradiated hairless mice with a significant enhancement of the antioxidant plasma capacity. Importantly, the antioxidant activity of PL was not lost during digestion but reached the bloodstream (increased oxygen radical absorbance capacity (ORAC), in plasma). Furthermore, the observed increase in superoxide dismutase (SOD), glutathione peroxidase (GP), and glutathione *S*-transferase (GST) activities cannot be explained as a function of increased expression as erythrocytes are anucleated [82]. Instead, it was postulated that PL may produce an allosteric activation of these enzymes. In the case of SOD, their activity decreased in both irradiated animals and vehicle-treated, and PL prevented such decrease of activity, but PL did not increase their expression, suggesting an activating modulatory effect.

A recent study on the mouse model reported the capacity of an orally administered PL extract to delay tumorigenesis induced by chronic exposure to UVR. PL also diminished the number of skin tumors in the surrounding non tumoral skin of animal. This effect correlated with significant changes in several oxidative stress markers in blood and skin, without changes in the expression of specific antioxidant enzymes. The observed photoprotective effect of PL can be due to its anti-oxidant properties and its capacity to produce allosteric changes in some enzyme systems [63].

Finally, PL complemented the effect of ascorbate and limited melanoma cell growth and the extracellular matrix (ECM) remodeling through, among other effects, by increasing the expression of TIMP-1 [65]. Oral administration of PL could offer significant photoprotective effects essential to the treatment and prevention of UV-induced skin cancer (Table 1).

### 3.7. Fernblock and Extracellular Remodeling: Collagen, Elastin, and Matrix Metalloproteinases Network

The remodeling of the ECM in cancer or skin aging is due to reduced biosynthesis of ECM and/or increased expression of matrix metalloproteinases (MMPs) [90], inhibition of collagen synthesis, or inhibition of TIMP. TGF-β is a primary regulator of ECM [91]. Aging skin displays lower levels of TGF-β, or reductions of its mediator, whereas TGF-β levels are enhanced in cancer. UV radiation reduces the microfibrillar network in the dermis and in the epidermal-dermal layer and contributes to the appearance of aberrant elastic fibers.

PL counteracts these alterations via its photoprotective, antioxidant and anti-inflammatory properties. PL exhibits a strong anti-aging effect. Additional anti-aging effects of PL include inhibition in vitro of the expression of MMP and increased expression of an endogenous metalloprotease inhibitor, TIMP. Inhibition of MMP-1 expression by PL in keratinocytes and fibroblasts has been reported [83]. In fibroblasts, PL inhibited the expression of MMP-2 and simultaneously stimulated TIMPs (TIMP-1 and TIMP-2). In vitro, in melanoma cells, PL preferentially inhibited MMP-1 in an AP-1-dependent manner, which is consistent with reduced degradation of interstitial collagen, and stimulated TIMP-2, implicated in the inhibition of basement membrane remodeling [78].

Regarding a direct effect on the ECM, PL stimulated deposition of types I and V collagen in UV-irradiated fibroblasts and types I, III, and V collagen in non-irradiated fibroblasts [78]. Its stimulatory effect on types I and V collagen was observed in both UVA- or UVB-irradiated fibroblasts, though UVB radiation decreased the level of stimulation of types I and V expression, and UVA radiation significantly counteracted the stimulation of collagen type I (COLIα1) promoter activity by PL. Conversely, UV radiation decreases the PL induction of type III collagen. These data imply that PL promotes the synthesis of types I and V collagen in UV radiation-exposed skin and the assembly of fibrillar collagens in the sun-protected skin. PL also modulates the expression of cytokines that control ECM remodeling and the biology of the cells implicated in this process. Specifically, PL promotes expression of TGF-β in non-irradiated or UV-irradiated fibroblasts but inhibits TGF-β in melanoma cells, which may be responsible for the observed inhibition of MMP-1 expression induced by in these cells. This effect may be responsible for the observed inhibition of angiogenesis in vivo [78]. In this regard, the effect of PL was largely similar to that of ascorbic acid.

Stimulation of collagen expression was associated with the increase of TGF-β, whereas UVB radiation-mediated inhibition of collagen synthesis correlated with decreased expression of TGF-β. However, UV radiation did not decrease PL stimulation of TGF-β expression, which suggests that UV and PL regulate TGF-β expression by separate, yet related, pathways [78].

In summary, PL protects the ECM through two types of actions: one depends on its effect on ECM proteolytic enzymes and the overexpression of TIMPs, and the second one is related to the expression/assembly of structural collagens (types I, III, and V), and TGF-β in fibroblasts. The anti-cancer effect of PL encompasses the inhibition of MMPs and stimulation of TIMPs, and decrease TGF-β in melanoma cells (Table 1).

### 3.8. Fernblock and VIS and IR Radiation

**Figure 2:**
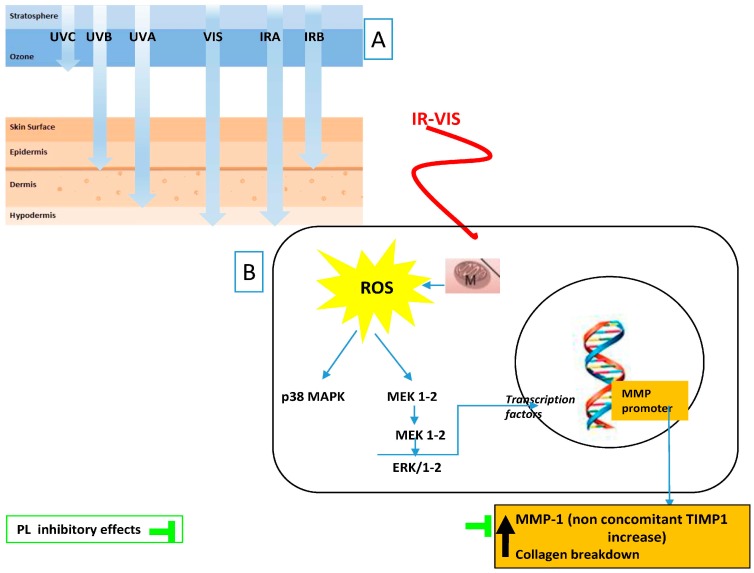
Tested anti IR–VIS effects of *Polypodium leucotomos* (PL) extract: (**A**) ultraviolet radiation (UV), visible light (VIS), and infrared radiation (IR) depth penetration in the skin; and (**B**) main PL effects reflect its capacity to decrease MMP-1 upregulation induced by cutaneous exposure to IR–VIS. Black arrows indicate increased/decreased effects due to IR–VIS.

The most recent attempt to palliate the above-mentioned deleterious effects of IR and VIS radiation was performed with Fernblock. The González group has shown its clinical efficacy in preventthe harmful effects of infrared-visible IR–VIS radiations [84] (Figure 2).

PL extract protects skin cells in vitro against IR–VIS radiations due to its antioxidant properties and to its ability to induce the repair of DNA damage. A prospective clinical trial has been performed, in which a gluteal biopsy from volunteers was performed before and after irradiating with IR–VIS (600 and 200 J/cm^2^, respectively). A mixture including Fernblock (960 mg/day) was administered for 21 days, and a new irradiation and biopsy were performed. Histological and molecular studies were performed to determine the levels of MMP1 before and after irradiation with and without treatment. MMP1 was increased after irradiation with VIS-IR with respect to baseline in 71% of the patients, while this increase significantly dropped (51.7%) in subjects treated with the oral photoprotector. Immunohistochemistry experiments revealed that irradiation did not significantly affect the structure of the epidermis [84] (Table 1).

## 4. Potential Use of Fernblok in the Treatment in Other Skin Pathology

### 4.1. Melanoma High-Risk Patients

Melanoma (MM) is a devastating disease, with high rate of metastasis. The constant increase in their incidence and chemotherapy resistance of MM has stressed the importance of its prevention. It is becoming increasingly clear that solar UV radiation is a major risk factor in the etiology of MM (and photoprotection is the only primary preventive method against its development).

Approximately 10% of MM occurs in a familiar context. Cyclin-dependent kinase inhibitor 2A (*CDKN2A*) and cyclin-dependent kinase 4 (*CDK4*) have been identified as MM susceptibility genes. It has been shown that 25% to 50% of familial MM kindred are affected by the mutation of *CDKN2A* [64].

Beyond genes known to be involved in a high degree of susceptibility to cutaneous MM, other genes have been proposed to confer moderate risk. Thus, variants in Melanocortin 1 receptor (*MC1R*) gene codified the synthesis of a red/yellow pheomelanin and induce oxidative cell damage [64].

A recent in vivo study has addressed the possible role of oral PL extract to increase photoprotection in patients with high risk of MM. First, that study described that PL improved systemic photoprotection by decreasing UV-induced erythema and increasing the minimal erythematous dose (UV-MED) in patients with patients with familial MM and sporadic MM, and dysplastic nevus syndrome. Although not significant, PL had a stronger effect on the MED in patients with familial MM than in sporadic MM. The study could also prove that patients with familial MM, and those exhibiting polymorphisms in *MC1R* and/or a mutated *CDKN2A* displayed the biggest difference in response to treatment with PL. According to this study, patients with lower basal MED (higher UVR sensibility) would benefit the most from oral PL treatment. The results are very promising, suggesting the need to perform new studies with long-term administration of PL in patients having a high risk of developing MM, and with long-term follow-up [64] (Table 2).

### 4.2. Idiopathic Photodermatosis

These lesions include clinical conditions that emerge after cutaneous exposure to sunlight. Some examples include solar urticaria, polymorphic light eruption (PMLE), actinic prurigo and chronic actinic dermatitis [92,93]. A recent study has addressed the PL potential to counteract the appearance of PMLE [93]. After two weeks of PL intake, it was observed a significant reduction in positive photoprovocation results and as well as a significant delay in PMLE lesions formation. Despite the small number of patients and taking into account that this was an open study, its results suggested use of PL extract in the prevention of PMLE in severely affected patients.

Other studies including patients affected with PMLE have reported the beneficial effects of PL. In those studies, patients exposed themselves to sunlight were treated with PL orally (480 mg/day). The response of the skin to sunlight exposure was compared to that occurring previously without PL. These studies displayed a relevant and significant reduction of skin reaction and improvement of subjective symptoms [94,95] (Table 2).

### 4.3. Actinic Keratosis

Actinic keratosis (AKs) is a common premalignant skin condition. Different of treatments are available, but photodynamic therapy (PDT) is one of the treatment most effectives for AKs. PDT could induce DNA-mutagenesis/immunosuppression, bearing to AKs therapeutic failure/recurrences. PL reduces immunosuppression induced by UV and mutagenesis. Patients who had at least two AKs on the scalp were subjected to two PDT sessions, one-week apart. The first group started oral treatment with PL one week after the last session of PDT. Both treatment modalities reduced the number of AKs. However, oral supplementation of PL added to PDT treatment increased clearance rate when compared to PDT alone and decreased recurrence rate of AKs within six months, supporting that oral PL may be used as a supplementary agent in the treatment of field cancerization [95].

### 4.4. Pigmentary Disorders

#### 4.4.1. Vitiligo

UVB phototherapy, narrow band (311–312 nm), is one of the most efficient treatment options for vitiligo vulgaris. The treatment promotes melanocyte reservoirs to counteract depigmentation. A double-blind, placebo-controlled study in patients with vitiligo showed that the use of PL and narrow-band UVB (NB-UVB) increases repigmentation of the affected head and neck area affected [96]. The observed effect might be attributable to the immunomodulatory and antioxidant properties of PL that would counter the possible autoimmune and/or oxidative origin of the disease.

Together with PL beneficial effect on PUVA-therapy [97], these studies suggest that PL may be general use in phototherapy protocols [98]. The addition of oral PL to NB-UVB for the treatment of vitiligo vulgaris shows a tendency toward increasing the amount of pigment in the neck and head areas that nearly reaches statistical significance. The repigmentation was more pronounced in patients with light skin types. In a second study, patients with generalized vitiligo were randomized to receive a therapy with both NB-UVB and PL (twice-weekly NB-UVB and oral PL 480 mg daily) for up to six months or NB-UVB phototherapy alone. The combination of PL and NB-UVB improved the re-pigmentation by 40% compared to 22% percent for NB-UVB alone. All patients treated with PL and NB-UVB showed significantly higher re-pigmentation [99] (Table 2).

#### 4.4.2. Melasma

Melasma, a common skin condition in adults, is an acquired hypermelanosis on sun-exposed areas of the skin. Several methods of treatment are available to patients with melasma; however, developing novel targeted therapies could help to understand the pathogenesis and improve the skin condition, which has important social and psychological ramifications [101,102].

A recent study has shown the efficacy of PL for treating melasma. Female patients with melasma were randomized to be treated for 12 weeks with oral PL or placebo twice daily [98]. Each patient applied sun protection factor (SPF) 45 sunscreen daily. The group of patients treated with PL had a significantly decreased in mean Melasma Area and Severity Index at 12 weeks of treatment with PL, whereas the placebo group did not [100] (Table 2).

### 4.5. Premature Aging

Skin aging is a time dependent event that results in changes in the appearance and the molecular composition of human skin. “Aging” should be seen as a combination of two biological processes: intrinsic or natural aging and the changes produced by the interaction of the skin with external environment or extrinsic aging. Extrinsic aging is mostly synonymous with photoaging because UV-radiation induces damage in exposed skin [103]. Human skin contains many antioxidant substances (redox-active) and enzymes that continually dampen the oxidative effect of the environment. If these substances are not present or malfunction, oxidation can have deleterious effects, including darkening, skin damage, and aging.

ROS affect nuclear DNA and also produce “common” medina deletion. As is well-known, these ROS-induced DNA alterations cause the generation 8-OH-dG. This mutation accumulates during aging [104]. UV irradiation leads to the formation of CPD. The capacity in repairing CPD clearly decreases with age [105]. This decrease in DNA damage repair capacity likely underlies the higher prevalence of skin cancer in older individuals. In addition, ROS are important factors in carcinogenesis and high levels of 8-OH-dG are found many types of cancers.

In aged skins, the alterations in the enzyme activities are not well characterized. However, disruption of Glutathione peroxidase 4 (GPx4), an enzyme implicated in antioxidative defense, displayed aging skin phenotypes, with an increase of lipid peroxidation, and with higher levels of the COX-2 [103].

In the context of aging, the disruption of the balance of the extracellular matrix plays an important role. The levels of skin collagen I, III and VII decrease in aging, which is exacerbated by the activation of MMPs. During the aging process, MMPs are upregulated but TIMP, are downregulated, with accelerated turnover ECM [106]. UV-irradiation increases the expression of ROS and activation of MAP-kinase, NF-κB and AP-1. Both AP-1 and NF-κB are important for the balance of proliferation and apoptosis, heavily involucrated in carcinogenesis in aged human skin. In addition to aging, UV-irradiation causes mutations in p53, which can lead to skin cancers [103]. In summary, PL protects against skin photoaging and oxidative damage due to the known molecular interaction with the intrinsic and extrinsic aging related factors (See Table 1).

## 5. Fernblock: A Road to (Present and Future) Prevention of UV-, VIS-, and IR-Mediated Skin Damage

Fernblock exhibits a wide array of beneficial effects revealing no significant toxicity or allergenicity. Its dual route of administration supports its preventive activity against skin photodamage, not only when given prior to sunlight exposure but also during exposure (Table 1, Figure 1 and Figure 2). It may also contribute to the cutaneous wound-healing process that is required post exposure with potential as an anti-aging and anti-cancer tool. Most of its beneficial effects are mediated by its antioxidant properties, both when administered topically and systemically. Recently, the positive effect of reducing IR–VIS damage has also been proposed. New evidence links PL to tumor development delay in mice, either by repairing the damaged DNA or by increasing apoptosis. Additional investigation will be addressed to evaluate its effects in other parameters related to photoaging and photocarcinogenesis.

## Figures and Tables

**Table 1:** Solar (ultraviolet (UV), infrared (IR), and visible light (VIS)) protection effects of *Polypodium leucotomos* (PL) and molecular target.

UV Effects Tissue/Cellular/Molecular Target	PL Counteracts UV-Effects	References
DNA damage	Inhibit of DNA mutations	[65,68]
	Inhibit accumulation of CPD	[65,68,76]
	Inhibit of 8-hydroxy-2′-deoxyguanosine	[65]
	Inhibit of mtDNA mutations	[31]
Inflammation	Inhibit of TNF-α, iNOS, NF-κB, AP-1, COX-2	[35,57,65]
	Decrease mast cell, neutrophils, and macrophage infiltration	[59,61,62]
	Inhibit of PUVA induced vasodilation	[71]
Immunosuppression	Inhibit of UVR-mediated Langerhans cell depletion	[67,68,77]
	Protect DCs from UV-induced apoptosis	[66,67,68]
	Induce DCs production of anti-inflammatory cytokines (IL-12)	[80]
	Reduce of glutathione oxidation in blood and epidermis	[66]
	Interfere the *cis*-UCA isomerization	[81]
Photo Carcinogenesis	Reduce the number of mice showing skin tumors at 8 weeks after the cessation of chronic UVB exposure	[75]
	Increase the number of p53(+) cells	[63,65,82]
	Increase TIMP	[65]
	Inhibit of angiogenesis	[77]
	Inhibit of epidermal cell proliferation	[68,76]
	Enhance the antioxidant plasma capacity	[71,82]
UV–ECM damage	Inhibit MMP-1 (also in melanoma cells)	[78,83]
	Increase TIMP (also in melanoma cells)	[78]
	Increase the synthesis of types I, III, and V collagen	[78]
IR–VIS Effects Tissue/Cellular/Molecular Target	PL Counteracts IR–VIS Effects	[84]
UV–ECM damage	Inhibit MMP-1	[84]

mtDNA, mitochondrial DNA; ECM, extracellular matrix; CPD, cyclobutane pyrimidine dimers; TNF-α, tumor necrosis factor-α; iNOS, isoforms of nitric oxide synthase; NF-κB, nuclear factor kappa beta; AP-1, activator protein-1; COX-2, cyclooxygenase-2 enzyme; PUVA, Psoralens + UVA; DC, dendritic cells; UVR, ultraviolet Radiation; UCA, urocanic acid; MMP-1, matrix metalloproteinase-1; TIMP, tissue inhibitor of metalloproteinase.

**Table 2:** Application of *Polypodium leucotomos* (PL) in the treatment of skin pathology.

Pathology	Potential Clinical Use of PL	References
Melanoma	PL extract improves systemic photoprotection in patients at risk of MM	[64]
	The strongest effect of PL in patients with familial MM, those exhibiting a mutated CDKN2A and/or polymorphisms in MC1R	[64]
Idiopathic Photodermatosis	PL significant reduces skin reactions and subjective symptoms.	[92,93,94]
Actinic Keratosis	PL improves PDT clearance and decreases AK recurrence rate at 6 months	[95]
Pigmentary Disorders	Vitiligo	[96,97,98,99]
	Addition of PL to the treatment with NB-UVB shows an increased repigmentation mainly in the head and neck area	
	Melasma	[100]
	PL had decreased Mean Melasma Area and Severity Index. Photographic assessment and patient self-assessments revealed mild and marked improvement by PL	
Aging	PL decreased the proposed skin aging oxidative damage	See Table 1

MM, melanoma; PDT, photodynamic therapy; AK, actinic keratosis; NB-UVB, narrow-band UVB.

## Abbreviations

8-OH-dG	8-Hydroxy-2′-deoxyguanosine
AP-1	Activator protein 1
COLIα1	Collagen type I
CD	Common deletion
COX-2	Cyclooxygenase-2
CPDs	Cyclobutane pyrimidine dimers
DC	Dendritic cells
eLC	Epidermal Langerhans cells
GP	Glutathione peroxidase
GPx4	Glutathione peroxidase 4
GST	Glutathione *S*-transferase
HPLC	High-performance liquid chromatography
NB-UVB	Narrow-band UVB
NF-κB	Nuclear factor kappa beta
PL	Polypodium leucotomos
PMLE	Polymorphic light eruption
PUVA	Psoralens + UVA
ROS	Reactive oxygen species
SOD	Superoxide dismutase
SSR	Solar simulated radiation
TGF-β	Transforming growth factor beta
Th1	T helper 1 lymphocyte
TIMP	Tissue inhibitor of metalloproteinase
TNF-α	Tumor necrosis factor-alpha
UCA	Urocanic acid
